# Angiogenic and Osteogenic Strategies for Skeletal Regeneration in Aging

**DOI:** 10.1007/s11914-026-00983-0

**Published:** 2026-09-09

**Authors:** Mariana Moraes de Lima Perini, Jiliang Li

**Affiliations:** Department of Biology, Indiana University, Indianapolis, IN 46202 USA

**Keywords:** Aging, Fracture Healing, Angiogenesis, Endothelial Progenitor Cells, Exosomes, STAT3

## Abstract

**Purpose of Review:**

Aging disrupts skeletal homeostasis, resulting in impaired fracture healing, increased risk of nonunion, and reduced regenerative capacity. These deficits are associated with dysregulated inflammation, impaired angiogenesis, and reduced osteogenesis, while current treatments remain limited in aged populations. This review examines emerging regenerative approaches that target both vascular and osteogenic dysfunction during skeletal repair.

**Recent Findings:**

Recent studies have emphasized the importance of angiogenic-osteogenic coupling in successful bone repair. Endothelial progenitor cells (EPCs) promote neovascularization, improve vascular function, and support osteogenesis, while EPC-derived extracellular vesicles (EV), particularly exosomes, enhance intercellular communication, angiogenesis, and tissue repair with fewer limitations than cell-based therapies. In addition, signal transducer and activator of transcription 3 (STAT3) has been identified as a key regulator of osteoblast function, angiogenesis, and bone remodeling, making it an attractive molecular target for enhancing skeletal regeneration.

**Summary:**

EPC-based therapies, exosome-mediated interventions, and modulation of STAT3 signaling are promising strategies to enhance skeletal regeneration by addressing both vascular dysfunction and impaired osteogenesis. Continued investigation into the mechanisms, safety, and translational potential of these approaches will be critical for advancing regenerative therapies for age-related skeletal disorders.

## Introduction

Bone fractures and skeletal degeneration represent a significant clinical burden, particularly in aging populations, where impaired bone quality and reduced regenerative capacity increase fracture risk and delay healing [1]. Bone fractures are common in the elderly and are associated with high morbidity, mortality, and healthcare costs [2]. Bone is a unique tissue that undergoes continuous remodeling through a balance between osteoblast-mediated bone formation and osteoclast-mediated bone resorption [3]. Through this process, bone has the capacity to regenerate without leaving behind any scar tissue, with the new bone being eventually indistinguishable from the original, uninjured bone [4]. However, despite advances in surgical repair and pharmacological support, 10% of fractures fail to heal or result in nonunions [5–11].

The U.S. Food and Drug Administration (FDA) defines nonunion as a fracture that is at least nine months old and for which there has been no signs of healing for at least three months [12, 13]. There are many factors that may put a patient at a higher risk for nonunions, with age and metabolic conditions, such as osteoporosis and diabetes, being some of the main issues [14, 15]. As aging is often accompanied by a decrease in bone quality, the aged population is at a greater risk of fractures and its associated morbidity, mainly due to the decreased activity of cells involved in the healing process of the bone [16, 17], resulting in more than 2.1 million age-related fractures a year in the U.S [2]. Understanding the molecular and cellular mechanisms that regulate skeletal homeostasis and regeneration, and how they are disrupted by aging, is essential to developing more effective treatments.

This review provides an overview of the cellular and molecular mechanisms regulating skeletal homeostasis, aging-associated bone decline, and emerging regenerative strategies aimed at improving bone repair. Particular focus is placed on therapeutic approaches targeting angiogenesis and osteogenesis, including endothelial progenitor cell-based therapies, extracellular-vesicle-mediated signaling, and STAT3 activation. Together, these approaches highlight the importance of vascular-osteogenic coupling in skeletal regeneration and represent promising strategies for enhancing bone healing in aging and compromised conditions.

## Skeletal Homeostasis, Aging, and Impaired Healing

### Bone Remodeling and Skeletal Homeostasis

Bone homeostasis is maintained through a tightly regulated balance between osteoblast-mediated bone formation and osteoclast-mediated bone resorption [3]. Disruptions in this balance, particularly with aging, result in decreased bone mass and increased osteoclast activity [1, 18]. Aging is associated with reduced osteoblast function, increased osteoclast activity, and altered signaling pathways that impair bone regeneration and remodeling [1, 18]. Understanding the mechanisms governing this balance is essential for developing therapies that improve both bone health and regenerative capacity.

### Stages of Fracture Healing

The successful healing of fractures depends on the biological environment of the fracture site and a mechanical environment that provides the fracture site with adequate stability [13]. It is a highly coordinated regenerative process involving overlapping phases of hematoma formation/inflammation, soft callus formation, hard callus formation, and bone remodeling [7, 13]. Immediately following injury, disruption of local vasculature leads to hematoma formation and hypoxic conditions, which stimulate the migration of osteogenic and angiogenic precursor cells to the site of injury [7, 19]. The formation of the hematoma results in the release of cytokines, such as tumor necrosis factor-alpha (TNF-$\:{\upalpha\:}$), bone morphogenetic proteins (BMPs), transforming growth factor-beta (TGF-$\:{\upbeta\:}$) superfamily, and interleukins [20, 21]. These cytokines stimulate the recruitment of inflammatory cells, such as macrophages, monocytes, and lymphocytes, that will act to remove damaged tissue and secrete other cytokines, such as vascular endothelial growth factor (VEGF) to induce healing and promote vascular proliferation [5, 13, 20, 22, 23]. The release of VEGF leads to the start of angiogenesis at the injury site. This is an important requirement for proper bone healing as adequate vascularization facilitates the delivery of oxygen, cells, and key nutrients to the fracture site [6, 20, 24] as well as provides multifunctional signaling molecules that regulate bone development, regeneration, and remodeling [25].

BMPs drive the differentiation of mesenchymal stem cells (MSCs) into fibroblasts, chondroblasts, and osteoblasts, which act together with endothelial cells (ECs) to give rise to a soft callus [7, 10, 20]. At this point, the vascular network has penetrated the cartilage template. RANKL expression further stimulates the differentiation of chondroblasts, chondroclasts, osteoblasts, and osteoclasts [20].

The soft callus will undergo secondary bone formation through both intramembranous and endochondral ossification [7]. In intramembranous ossification, MSCs will differentiate into osteoblasts, which will form bone through an anabolic process. In endochondral ossification, MSCs differentiate into chondrocytes, which form cartilage tissue. Subsequently, osteoblasts mineralize the cartilage template, leading to the progression from a soft callus to a hard callus [7]. Angiogenesis plays a crucial role in both intramembranous and endochondral ossification [20, 24]. It allows for the migration of more MSCs, osteoblasts, osteoclasts, and provides the appropriate microenvironment for the callus to undergo repeated remodeling. Remodeling can last for many months, until the bone has regenerated to its original structure [15, 20, 26].

### Impacts of Aging in Healing

The reduced regenerative capacity of aged bone has been attributed to multiple factors, including reduced numbers of stem and progenitor cells, impaired cell migration and proliferation, altered revascularization, and changes in tissue material properties [1, 18, 27]. Similar impairments are also observed in metabolic conditions, such as obesity and diabetes [1]. The mechanical properties of bone, including elasticity, strength, and resistance to failure, determine how it responds to mechanical loads and withstands stress, impacting both fracture risk and healing potential [28]. With age, bone becomes less stiff and more brittle, reducing its ability to withstand force and stress, thus making it more susceptible to fractures [29].

Delayed fracture healing in aged individuals is thought to be due to the disruption of the three main processes of the healing process: Inflammation, angiogenesis, and osteogenesis. Aging is often accompanied by chronic and dysregulated inflammation, leading to a delay in resolution and repair of the injury. Further, stem cell senescence, the lower capacity of MSCs to differentiate into osteoblasts, favoring adipogenic differentiation instead, combined with decreased osteoblast function, impaired angiogenesis and vasculogenesis through reduced VEGF expression and EPC mobilization, lead to a slower, less robust fracture healing with an increased risk of nonunions [30, 31].

VEGF, aside from being one of the most important regulators for vascular development and angiogenesis, plays critical roles in skeletal development [9]. Consistent with its role in skeletal development, VEGF also plays a critical role during fracture healing [9]. In the context of fracture healing, VEGF stimulates angiogenesis, directly influences bone formation, and induces osteoblast differentiation and proliferation [7]. VEGF production is stimulated by hypoxia, including during fracture healing [9, 32]. Studies conducted in older animal models show reduced VEGF production during fracture healing compared to young animals. This is attributed to the decreased activation of hypoxia-inducible factor 1a (HIF-1a), a transcription factor essential for the regulation of VEGF expression in response to hypoxia or ischemia [33, 34]. A comparison of angiogenic gene expression levels between mice of 2.5 months of age, 6 months of age, and 22 months of age showed that the mRNA levels of VEGF and Flt-1 were lower in 22-month-old animals in comparison to the younger ones [35]. Flt-1 is a cell-surface receptor for VEGF and plays a crucial role in the proliferation and survival of ECs and in the regulation of angiogenesis and cell survival and migration [35]. This reduction may contribute to the age-dependent impairment of angiogenesis and fracture healing observed in aged animals. Aged ECs will usually become flatter and enlarged, leading to changes in cytoskeleton integrity, angiogenesis, proliferation, and cell migration [36]. In addition to hypoxia, VEGF expression is also controlled by a cascade of cellular signaling events and transcription factors, including signal transducer and activator of transcription factor 3 (STAT3) [37, 38].

STAT3 regulates a variety of cellular events, including proliferation, differentiation, and apoptosis [32, 39]. It transduces signals of cytokines and growth factors, including VEGF, from the activated receptors to the nucleus to activate transcription by binding specific DNA motifs [37, 40, 41]. By transducing VEGF signals, STAT3 promotes EC proliferation, migration, and survival [37].

In a study conducted by Chang et al., it was found that aged ECs under hypoxic conditions exhibited significantly impaired proliferation, migration, and tube formation when compared with young cells. Furthermore, they observed that Stat3 was significantly downregulated in the aged cells and in the tissue of limbs of aged mice [42]. These findings suggest that age-related impairment in endothelial cell function may be partially driven by reduced STAT3 signaling, highlighting its potential role as a key regulator of angiogenesis and vascular-mediated bone repair.

## Limitations of Current Therapies

One important constituent for proper bone healing is the extracellular matrix that provides a natural scaffold for cellular events and interactions that occur during the healing process. In the clinical setting, there are various osteoconductive materials that serve as scaffolds, which can be utilized alone or in a combination with osteogenic, osteoconductive, and osteoinductive factors to promote healing [43, 44]. Osteogenesis requires angiogenesis as well as ingrowth of MSCs and osteoblasts [43, 44]. Osteoconduction refers to the ability of the graft to provide a scaffold in which bone can grow [45]. Osteoinduction describes the graft’s ability to recruit MSCs and stimulate their differentiation into chondroblasts and osteoblasts, a process that requires the presence of many growth factors [45].

### Bone Grafts

Bone grafting remains a standard strategy to enhance bone healing, with autografts considered the clinical gold standard due to their osteogenic and osteoinductive properties. However, autografts are limited by donor-site morbidity, limited tissue availability, and additional surgical complications [44, 46–49]. Complications involving bone harvesting for autograft have been reported in around 20.6% of cases [50]. Bone allograft, on the other hand, does not require further surgeries, since cadaver bones are used, but may transmit infectious diseases or lead to immune rejections [47, 51]. Moreover, the sterilization and storage processes of the donated bone may result in changes in the biological and mechanical properties of the tissue, leading to the loss of osteoinductive or osteogenic capabilities [50, 52].

Synthetic bone substitutes have been developed as promising alternatives [4, 47, 48]. However, many lack sufficient angiogenic potential, limiting their efficacy in large defects and aged patients with impaired vascularization [46, 50].

### Bone Morphogenetic Proteins (BMPs)

One commonly used method to accelerate the bone repair is to increase the local availability of BMP-2. BMPs are members of the TGF-$\:{\upbeta\:}$ superfamily [53]. They are the most common active biological substances used for bone healing and regeneration as they play a crucial role in the development of many tissues, including the skeleton. They have the ability to induce bone formation by both osteoconduction and osteoinduction, as has been demonstrated in various studies [49]. The administration of BMP-2 is done by the localized application to the fracture site [30]. Even though its use is FDA approved and it has been proven successful in the treatment of open tibial fractures, spinal fusion surgeries, and oral maxillofacial reconstruction procedures, BMP-2 as a treatment option does come with limitations [30, 54–57]. The use of BMP-2 as a treatment for fractures requires that the fracture surface be exposed, and if that is not possible, those fractures cannot be treated with BMP-2. Furthermore, continuous administration of BMP-2 is not feasible as the fracture is only exposed during initial surgery [30]. Since its approval by the FDA, the increasing use of BMP-2 has led to an increased side effects profile, not surprisingly, since BMP-2 significantly affects adipogenesis, inflammation, and osteoclast activation [56]. Some of the adverse effects that accompany the use of BMP-2 in the treatment of fractures are ectopic bone formation, formation of bone cysts, inflammation, and tumor growth [56, 58–60].

### Parathyroid Hormone (PTH)

Parathyroid hormone (PTH) is an anabolic agent that enhances osteoblast activity, increases bone mineral density, and improves bone healing [61, 62]. Although PTH has demonstrated beneficial effects in fracture repair, concerns regarding hypercalcemia, osteosarcoma risk observed in animal studies, treatment cost, and the need for prolonged daily injections have limited its widespread clinical application [30, 61–63]. Despite its broad orthopaedic applications, further research is needed to establish its efficacy and therapeutic value in fracture healing [61, 62, 64].

### Platelet Derived Growth Factor (PDGF)

Platelet-derived growth factors (PDGFs) are a family of dimeric growth factors (PDGF-AA, -AB, -BB, -CC, and -DD) that regulate cell proliferation, migration, and angiogenesis [65]. In bone, PDGF-BB is the most commonly used member of the PDGF family as it is a key regulator in tissue repair and regeneration for its ability to activate multiple PDGF receptors, such as PDGFR-$\:{\upalpha\:}$ and PDGF-$\:{\upbeta\:}$ [66]. Recombinant human PDGF-BB (rhPDGF-BB) combined with $\:{\upbeta\:}$-tricalcium phosphate ($\:{\upbeta\:}$-TCP) is currently approved by the FDA to aid in bone healing in ankles [67]. Different medical devices have incorporated PDGF-BB into other biomaterials for improved bone healing, but its use does not always improve bone healing. Furthermore, preclinical studies demonstrate that PDGF-BB enhances angiogenesis and stimulates the recruitment of MSCs and osteoprogenitor cells [68]. However, despite these pro-angiogenic effects, PDGF has been shown to antagonize the effects of BMP-2 and interfere with osteogenesis by impairing osteogenic differentiation and reducing bone formation when used in combination. Thus, the efficacy of PDGF is still being questioned in the bone healing process [66, 67]. Moreover, most clinical trials focus on foot and ankle surgery, with limited data in osteoporotic and aged populations. Overall, PDGF-BB is a promising but controversial option, with efficacy in fracture healing still under debate.

### Bone Marrow Aspirate Concentrate (BMAC)

Bone marrow (BM) aspirate concentrate (BMAC) has emerged as a regenerative strategy for delayed unions and nonunions due to its enrichment of MSCs and growth factors involved in osteogenesis and angiogenesis [69–72]. Studies suggest that BMAC may improve bone healing and reduce union time; however, its therapeutic mechanisms remain incompletely understood [70, 73]. Furthermore, variability in cell composition, lack of standardized harvesting protocols, and inconsistent clinical outcomes continue to limit its widespread application [72, 74, 75].

### Stem Cell Therapies

Stem-cell based strategies aim to restore the osteogenesis/angiogenesis coupling for proper fracture healing but face translational constraints in the elderly. Many drawbacks accompany stem cell therapies, such as senescence and diminished function of endogenous and transplanted cells, poor survival/engraftment in hypoxic and inflamed fracture niches, immunogenicity, tumorigenicity risks, and manufacturing/regulatory hurdles [76–79]. These limitations have motivated increasing interest in cell-free regenerative approaches, including EVs and exosomes.

## Emerging Therapies for Fracture Healing in Aging

### Endothelial Progenitor Cells

Bone fractures accompanied by vascular injury exhibit substantially impaired healing, with complication rates reported to be as high as to 46% [30, 80, 81]. In the past, studies of bone regeneration focused solely on the implantation of scaffolds and transplantation of MSCs [82]. However, advancements in vascular biology have highlighted that angiogenesis is indispensable for effective fracture repair and overall bone health [82].

Within the stem cell population, endothelial progenitor cells (EPCs) have emerged as an attractive candidate population due to their central role in vascular homeostasis, endothelial regeneration, neovascularization, angiogenesis, and vascular repair [83, 84]. A study using an angiogenesis inhibitor, TNP-470, on the healing of closed femoral fractures in a rat model, showed that inhibiting angiogenesis completely prevented fracture healing. The formation of a fracture callus and the deposition of periosteal woven bone were both suppressed, thereby disrupting both intramembranous and endochondral ossification. This underscores angiogenesis as an essential step of the early stages of fracture healing [85].

EPCs were first characterized in 1997 as bone marrow-derived progenitors capable of differentiating into mature endothelial cells and incorporating into the foundation of neovasculature [86, 87]. Upon specific stimuli, such as injury or hypoxia, EPCs can mobilize from the bone marrow, migrate to sites of tissue damage, and contribute to vascular repair either through direct endothelial differentiation or via paracrine signaling. Through the secretion of extracellular vesicles (EVs), including growth factors, cytokines, chemokines, and lipids, EPCs enhance reendothelialization, recruit additional progenitors, and support angiogenesis [83, 87–89]. Their angiogenic potential, combined with a lower risk of ectopic tissue formation compared to MSCs (Table 1), has contributed to growing interest in their therapeutic use in fracture repair [6, 82, 90].

**Table 1:** Comparison of EPCs and MSCs in Bone Regeneration

Feature	Endothelial Progenitor Cells (EPCs)	Mesenchymal Stem Cells (MSCs)
**Primary Role**	Angiogenesis, endothelial regeneration, and vascular repair	Osteogenesis, chondrogenesis, and stromal support
**Main Source**	Bone marrow, peripheral blood, and umbilical cord blood [91, 92]	Bone marrow, adipose tissue, and periosteum [93]
**Key Mechanisms**	Differentiation into endothelial cells; paracrine release of EVs (growth factors, cytokines, microRNAs) [92]	Differentiation into osteoblasts/chondrocytes; paracrine support of bone remodeling
**Commonly Reported Markers**	CD31, VE-cadherin, VEGFR1/2 [92, 94]	CD73, CD90, CD105 (positive); CD34/CD45 (negative) [95–97]
**Contribution to Healing**	Restores vascularization at fracture site; enhances nutrient delivery and osteoprogenitor recruitment [98]	Direct bone-formation capacity; modulates immune environment [96, 98]
**Age-Related Decline**	Reduced number and function with aging but retain some angiogenic potential [99]	Strongly affected by senescence: reduced self-renewal, impaired differentiation, weaker paracrine signaling [93, 96]
**Advantages**	Targets vascular deficit in elderly fractures; promotes both angiogenesis and osteogenesis indirectly; less risk of ectopic ossification [6, 30, 100]	Direct osteogenic capacity; well-studied in bone repair; easier expansion in vitro [96, 97]
**Limitations**	Limited clinical data in fracture repair; cell sourcing and phenotypic stability concerns; low numbers in elderly patients [30, 99]	Senescence in aged donors; risk of ectopic tissue formation; variable engraftment [101–103]
**Current Applications**	Clinical trials in ischemic diseases (stroke, myocardial infarction, diabetic foot, liver cirrhosis) [89, 104]	Clinical trials in bone regeneration, cartilage repair, spinal fusion [105, 106]

EPCs are commonly identified using combinations of endothelial and progenitor markers, although no universal phenotypic definitions exist. Frequently reported markers include CD31, CD34, VE-cadherin, VEGFR1, and VEGFR2 [83, 84]. VEGF is a key regulator of EPC mobilization and differentiation, and its essential role in bone healing has been well established [107, 108]. VEGF inhibition has been shown to impair cortical bone defect repair in a mouse model [109], while exogenous VEGF applied to the fracture site improved blood vessel formation, ossification, and callus maturation in mouse femur fractures and promoted callus bridging on a rabbit radius segmental gap defect [109]. Notably, aging diminishes vascular supply to fracture sites, often leading to delayed healing [2]. Mechanistically, aged and diabetic mice display reduced VEGF expression, delayed inflammatory resolution, and impaired chondrogenesis, partly due to elevated TNF-$\:{\upalpha\:}$ levels [2].

EPC interactions with other bone-regenerative cells have also been investigated. Co-culture studies demonstrate that EPCs can enhance the osteogenic differentiation of MSCs, shown by increased alkaline phosphatase (ALP) activity and matrix mineralization [90, 110]. Similarly, EPCs promote osteoblast function and contribute to hematopoietic stem cell maintenance [111]. Altogether, these findings suggest that EPCs not only stimulate angiogenesis but also create a pro-regenerative microenvironment conducive to bone formation. Importantly, age-related declines in the number and functionality of EPCs may explain the impaired angiogenic potential within the fracture site and delayed fracture healing often observed in the elderly population [111].

In vivo studies further support EPCs as a therapeutic strategy. Local delivery of EPCs seeded on biomaterial carriers, such as gel foam sponges [86], type I collagen scaffolds [112], or hydroxyapatite and tri-calcium phosphate (HA/TCP) scaffolds [112], has consistently improved neovascularization, callus formation, and new bone deposition in rodent models of segmental defects and fractures. More recent work has demonstrated that EPC-mediated bone regeneration can be achieved using multiple delivery vehicles, including phosphate-buffered saline (PBS), suggesting that simple, non-immunogenic delivery methods may facilitate future clinical translation [113]. More recently, neonatal EPCs delivered on collagen scaffolds enhanced fracture repair in aged mice, leading to increased bone volume and higher density compared with controls [6].

Clinically, the use of EPC-based therapies is being investigated in human clinical trials for conditions such as ischemic stroke, myocardial infarction, liver cirrhosis, and diabetic foot ulcers [30]. However, limited clinical evidence exists in the context of fracture healing, and almost no studies have been performed specifically in aged or metabolically compromised patients. Recent preclinical studies have begun to address several important translational barriers. Dose-response studies demonstrate that higher EPC doses improve radiographic union, bone volume, and biomechanical strength, emphasizing the importance of optimizing treatment parameters for clinical translation [114]. Likewise, cryopreservation studies have shown that EPCs retain their regenerative capacity following storage, supporting the feasibility of standardized, off-the-shelf cell products [115]. Despite these advances, challenges remain, including ensuring consistent EPC sourcing, stable phenotypic characterization, and minimizing risks such as immune rejection, tumorigenesis, and ectopic tissue formation [83]. To address these concerns, cell-free therapies are an innovative therapeutic approach [87]. EPC-Exos and conditioned media retain the paracrine regenerative capacity of EPCs while avoiding many of the challenges of cell transplantation. Altogether, EPCs represent a promising therapy for fracture healing by coupling angiogenesis with osteogenesis. Collectively, these studies have begun to define important translational parameters for EPC-based therapies. Future research must now prioritize validation in aged and metabolically compromised models, along with well-designed clinical trials to advance clinical translation.

### EPC-Derived Exosomes

Interest in EV-based therapeutics has increased substantially in recent years due to their potential to treat a wide range of pathological conditions, including musculoskeletal diseases. EVs are membrane-bound particles released by nearly all cell types and are generally classified into three major subtypes: microvesicles, apoptotic bodies, and exosomes [83, 116]. Microvesicles (100–1000 nm) originate from outward blebbing of the plasma membrane. Apoptotic bodies (400–1000 nm) are produced and released during the late stages of apoptosis [83, 84, 116]. In contrast, exosomes are lipid bilayer nanoparticles (30–150 nm) secreted into the extracellular environment by most eukaryotic cells via multivesicular body fusion with the plasma membrane [117–120].

Exosomes are formed through invagination or endocytosis and are enriched in bioactive cargo, including lipids, proteins, and nucleic acids (mRNAs and miRNAs) [119, 121–123]. These molecules mediate intercellular communication and have been shown to regulate bone homeostasis, skeletal development, and fracture healing through both paracrine and autocrine mechanisms [88, 120, 124]. Common biomarkers used to identify exosomes include CD9, CD63, CD81, TSG101, and ALIX [125]. Functional studies have proposed that recipient cells can internalize exosomal miRNA in order to regulate gene expression, cellular behavior, and their morphology [123, 124]. Importantly, exosomes have been shown to regulate tissue regeneration by suppressing apoptosis, modulating immune responses, maintaining endogenous stem cell niches, and stimulating angiogenesis [126].

Unlike direct cell transplantation, exosomes exhibit reduced immunogenicity and tumorigenic risk, while retaining many of the paracrine regenerative functions of their parent cell [120]. As such, bone marrow mesenchymal stem cell-derived exosomes (BMSC-Exos) have shown considerable promise in enhancing bone repair. Studies demonstrate that BMSC-Exos promote endogenous angiogenesis, myogenesis, and osteogenesis, possibly through the activation of osteogenic signaling cascades such as the BMP-2/Smad1/Runx2 pathway [117]. In a rat model of femoral nonunion, the administration of BMSC-Exos significantly increased the expression levels of angiogenesis-related genes, such as VEGF and HIF-1a, and visibly improved angiogenesis [117]. Similarly, BMSC-Exos enhanced the proliferation and osteogenic differentiation of older BMSCs in bone regeneration in aged rats [120, 127]. Collectively, these studies support the therapeutic potential of exosomes for enhancing fracture healing in the elderly population.

While MSC-derived exosomes have been extensively investigated for skeletal regeneration, comparatively fewer studies have evaluated endothelial progenitor cell-derived exosomes (EPC-Exos) in bone repair. Beyond MSCs, EPC-Exos have recently emerged as promising mediators of vascular repair and potential cell-free therapeutic candidates for skeletal regeneration. Evidence suggests that the paracrine activity of EPCs may be more important than their direct incorporation into neovasculature, with EPC-Exos serving as the principal effectors [128]. In addition to promoting angiogenesis, EPC-derived EVs have been shown to regulate bone marrow stromal cell proliferation and osteoblastic differentiation, further supporting their role in angiogenic-osteogenic coupling [129]. It has been reported that EPC-Exos promote angiogenesis in diabetic wound healing and accelerate re-endothelialization after vascular injury in rats [128, 130]. Another study evaluating the effects of EPC-Exos on skin wound healing of diabetic mice showed that the healing process was accelerated with the use of EPC-Exos and confirmed by the increased expression of angiogenesis-related factors, including VEGF and CD31, and cell proliferation marker Ki67 [131]. In vitro, EPC-Exos increased endothelial cell proliferation, migration, and tube formation [132], and in vivo administration improved carotid artery re-endothelialization by augmenting endothelial proliferation and migration [123]. Importantly, direct preclinical evidence supporting the bone regenerative potential of EPC-Exos has also emerged. In a rat distraction osteogenesis model, EPC-Exos enhanced angiogenesis and bone regeneration, resulting in enhanced bone formation and improved biomechanical properties, supporting their potential as a cell-free strategy for skeletal repair [130]. Despite these encouraging findings, direct evidence supporting the use of EPC-Exos for skeletal regeneration remains limited. Compared with MSC-derived exosomes, relatively few studies have specifically evaluated EPC-Exos in bone repair, and much of the current evidence is derived from models of vascular injury or diabetic wound healing. Therefore, while EPC-Exos represent a promising cell-free therapeutic strategy, additional studies are needed to define their mechanisms of action, optimize therapeutic cargo, and establish efficacy in clinically relevant models of age-related fracture repair. Furthermore, studies investigating EPC-Exos in aged animal models remain scarce, highlighting an important gap in the literature and an opportunity for future research in age-related fracture healing.

Although isolation methods, dosing requirements, and standardization challenges remain limitations for clinical translation, exosomes confer several advantages over cell-based therapies [120]. Exosomes are non-living vesicles enriched in proteins and RNAs, can be engineered to carry therapeutic cargo, remain stable under physiological conditions, and generally exhibit lower immune responses or malignant transformations than cell-based therapies [117, 120, 127]. These characteristics make exosomes attractive candidates for cell-free regenerative therapies. However, challenges related to standardized manufacturing, quality control, and scalable production remain major barriers to clinical translation [100]. Additionally, exosomes appear to act at multiple important steps for tissue restoration, such as inflammation, fibrosis, and vascular EC proliferation and migration, suggesting that exosome-based therapies and cell-based therapies may be complementary to each other [123].

A critical question that remains largely unanswered is how aging influences exosome biogenesis, cargo composition, and functional potency. Given that aging is associated with cellular senescence, impaired angiogenesis, and increased marrow adiposity, understanding age-related alterations in exosomal signaling could provide novel insights into skeletal aging and lead to targeted rejuvenation and restoration strategies [116]. Future studies should also focus on optimizing exosome isolation methods, cargo characterization, dosing strategies, and targeted delivery systems while validating therapeutic efficacy and long-term safety in aged fracture models.

### Signal Transducer and Activator of Transcription 3

STAT3, in its inactive form, is highly expressed in the cytoplasm in most cell types. It is activated by a wide range of signaling pathways, including cytokines from the IL-6 family and growth factors, such as PDGF [133]. STAT3 plays a central role in both angiogenesis and osteogenesis, as well as in the regulation of the immune system [134]. Mutations in the STAT3 gene lead to Job’s Syndrome, a rare immunodeficiency disease characterized by skeletal abnormalities including craniofacial defects, reduced BMD, and recurrent fractures [135, 136]. Because the inflammatory phase is critical for successful fracture healing, dysregulation of inflammatory signaling can impair angiogenesis, callus formation, and bone remodeling. Given the central role of STAT3 in coordinating immune responses, these findings suggest that STAT3 may represent a promising therapeutic target for delayed union and nonunion [137]. Supporting this concept, a clinical study examining the STAT3-mediated regulation of immunity in adult patients with closed tibia fractures found that patients with early fracture healing exhibited significantly higher STAT3 expression than those with delayed healing, indicating a beneficial role of STAT3 in the bone healing process [134].

Activation of STAT3 in MSCs and ECs has been shown to promote proliferation and differentiation, thereby enhancing bone repair [138]. Because STAT3 regulates both endothelial and osteogenic cell function, it represents a unique molecular target capable of simultaneously promoting angiogenesis and osteogenesis during fracture repair. Our group has previously shown that inactivation of Stat3 in osteoblast/osteocyte in mice results in lower BMD, reduced bone strength, and a diminished response to ulnar mechanical loading, indicating that Stat3 is essential for bone maintenance and mechanotransduction [139]. Osteoblast-specific deletion of Stat3 leads to skeletal defects, including osteoporosis and spontaneous bone fractures. Conversely, pharmacological activation of STAT3 partially rescued these defects, whereas inhibiting Stat3 aggravated bone loss [140]. In a study by Yadav et al., in which Stat3 function was deleted from the developing limb mesenchyme or osteoprogenitor cells in mice, it was found that the resulting shortened and bow limbs resembling the symptoms of Job’s Syndrome were due to a down-regulation of Wnt/$\:{\upbeta\:}$-catenin signaling [40]. Many Wnt ligands and receptors as well as $\:{\upbeta\:}$-catenin protein are upregulated during the entire fracture healing process, inducing osteogenesis [19, 40].

Several studies have shown that increasing activation of STAT3 in MSCs promotes osteogenic differentiation and enhances bone defect healing [141]. More recent evidence supports the therapeutic relevance of STAT3 signaling in skeletal regeneration, demonstrating that, in addition to promoting osteogenic differentiation, STAT3 serves as a central regulator of angiogenic-osteogenic coupling by coordinating endothelial and osteogenic responses during bone repair. Collectively, these findings support the therapeutic potential of STAT3 for enhancing fracture healing, particularly under conditions of impaired regeneration [142]. Cellular hypoxia has also been shown to enhance the osteogenic differentiation of MSCs and promote bone repair in femoral defects, coinciding with increased VEGF and STAT3 expression both in vivo and in vitro [19]. Inhibition of STAT3 signaling reverses these effects, suggesting that STAT3 may be implicated in VEGF-mediated angiogenesis and bone repair [19]. Recent studies emphasize that fracture healing recapitulates developmental signaling pathways regulating skeletal stem and progenitor cell fate, a concept that is consistent with the role of STAT3 in coordinating regenerative responses during bone repair [143].

STAT3 is broadly associated with angiogenesis under both physiological and pathological conditions [41]. In particular, the constitutive activation of STAT3 signaling has been highly correlated with tumorigenesis, in part through its ability to upregulate VEGF expression and in turn induce tumor angiogenesis [41, 144]. Importantly, therapeutic strategies will likely require tissue-specific and temporally controlled STAT3 activation rather than systemic constitutive activation. In contrast, unpublished data from our group indicate that constitutive Stat3 activation in osteoblasts does not induce tumor formation; rather, it significantly enhances lamellar bone formation, highlighting a bone-specific anabolic effect of STAT3 signaling. Recent comprehensive reviews further support STAT3 as a central regulator of bone development, remodeling, mechanotransduction, and repair, highlighting its role in coordinating osteogenic and angiogenic responses across multiple skeletal cell populations [145]. Collectively, these findings suggest that therapeutic modulation of STAT3 may represent a strategy to simultaneously enhance angiogenesis and osteogenesis while preserving normal skeletal homeostasis. Nevertheless, additional studies are needed to determine the long-term safety, optimal timing, tissue-specific regulation, and therapeutic dosing of STAT3 activation before clinical translation can be realized.

## Conclusions

Age-related decline in skeletal homeostasis presents significant clinical challenges, including reduced bone mass, increased fragility, and impaired regenerative capacity due to impaired angiogenesis, reduced osteogenic potential, chronic inflammation, and the accumulation of senescent cells. Current clinical interventions, such as bone grafts, BMPs, PTH, PDGF, and bone marrow aspirates, have demonstrated some success, but their limitations, including donor-site morbidity, immunogenicity, high costs, and other adverse effects, restrict their applicability in elderly patients. These shortcomings highlight the urgent need for more targeted, safe, and effective regenerative therapies [16, 77, 146].

Emerging approaches offer promising solutions by addressing both the vascular and osteogenic deficits of aging bone repair. EPCs and EPC-Exos represent promising therapeutic strategies by restoring angiogenesis and coupling vascular and skeletal regeneration while minimizing the limitations associated with conventional cell-based therapies. Modulation of STAT3 signaling has been shown to enhance both osteogenesis and angiogenesis, positioning it as a promising molecular target for improving bone healing in aged tissues (Table 2).

**Table 2:** Summary of Emerging Therapeutic Strategies for Enhancing Bone Repair

Feature	EPCs	STAT3	EPC-Exosomes
**Target Cell**	ECs and osteoblasts	ECs and osteoblasts	ECs and osteoblasts
**Mechanism**	Angiogenesis, homing to injury sites	Signaling hub, enhancing osteogenesis and angiogenesis, regulating immune responses	miRNA and proteins delivery to target cells
**Aging Effect**	Decline in number/function limiting reparative capacity	Reduced activation, weakening pro-healing effects	Can compensate for age-related declines, as can be sourced from healthy donors
**Healing Outcome**	Improved angiogenesis and bone repair in preclinical models, but limited by age-related decline	Enhanced angiogenesis and osteogenesis in preclinical models	Promising cell-free regenerative strategy with encouraging preclinical evidence

Future studies must focus on overcoming key translational barriers. For EPC- and exosome-based therapies, standardized isolation, characterization, and delivery methods are necessary to improve reproducibility, scalability, and clinical safety. In addition, understanding how aging alters exosome biogenesis and cargo composition will be critical for optimizing their therapeutic efficacy. For STAT3-targeted therapies, further mechanistic studies in aged animal models are needed to evaluate efficacy while minimizing oncogenic risks or off-target effects. Combinatorial approaches, such as coupling exosome delivery with STAT3 activation, may ultimately prove more effective than single-agent strategies by simultaneously addressing multiple biological processes involved in fracture repair.

In the coming years, well-designed preclinical studies and clinical trials that account for the biological complexities of aging will be essential to advance these therapies into clinical practice. By integrating vascular biology, bone cell signaling, and biomaterial-based delivery platforms, the next generation of regenerative approaches holds strong promise for reducing fracture-related morbidity and mortality in the elderly population. Together, these emerging therapeutic strategies represent a shift from replacing lost bone toward restoring the coordinated vascular and osteogenic processes required for successful skeletal regeneration, particularly in the aging population.

## Key References

- Saul, D. and S. Khosla, *Fracture Healing in the Setting of Endocrine Diseases, Aging, and Cellular Senescence.* Endocr Rev, 2022. 43(6): p. 984-1002.
  - ○ This review summarizes the effects of aging, endocrine diseases, and cellular senescence on fracture healing. The authors discuss how age-related changes in inflammation, angiogenesis, and osteogenesis contribute to delayed healing and an increased risk of nonunion, highlighting the potential therapeutic targets for improving skeletal regeneration in older adults.
- Shi, H., et al., *A Review Into the Insights of the Role of Endothelial Progenitor Cells on Bone Biology.* Front Cell Dev Biol, 2022. 10: p. 878697.
  - ○ This review summarizes the effects of aging, endocrine diseases, and cellular senescence on fracture healing. The authors discuss how age-related changes in inflammation, angiogenesis, and osteogenesis contribute to delayed healing and an increased risk of nonunion, highlighting the potential therapeutic targets for improving skeletal regeneration in older adults.
- Hu, S., et al., *Exosomes promise better bone regeneration.* Regen Ther, 2025. 30: p. 389-402.
  - ○ This review summarizes the effects of aging, endocrine diseases, and cellular senescence on fracture healing. The authors discuss how age-related changes in inflammation, angiogenesis, and osteogenesis contribute to delayed healing and an increased risk of nonunion, highlighting the potential therapeutic targets for improving skeletal regeneration in older adults.
- Jia, Y., et al., *Exosomes secreted by endothelial progenitor cells accelerate bone regeneration during distraction osteogenesis by stimulating angiogenesis.* Stem Cell Res Ther, 2019. 10(1): p. 12.
  - ○ This study demonstrated that endothelial progenitor cell-derived exosomes enhance bone regeneration by promoting angiogenesis during distraction osteogenesis. The authors highlight the therapeutic potential of EPC-derived exosomes as a cell-free approach for skeletal repair.
- Chen, L., et al., *STAT3 activation by catalpol promotes osteogenesis-angiogenesis coupling, thus accelerating osteoporotic bone repair.* Stem Cell Res Ther, 2021. 12(1): p. 108.
  - ○ This study demonstrated that activation of STAT3 enhances osteogenesis and angiogenesis during bone repair, resulting in improved healing in an osteoporotic model. The authors highlight the role of STAT3 in angiogenic-osteogenic coupling and its potential for improving skeletal regeneration..

## Data Availability

No datasets were generated or analysed during the current study.
